# Exploring the characteristics of the sibling relationship in vision impairment in developmental age: a systematic review

**DOI:** 10.3389/fpsyg.2025.1652978

**Published:** 2025-10-29

**Authors:** Tiziana Battistin, Alessia Zanatta, Vincenzo Zanardo, Maria Eleonora Reffo, Elena Mercuriali

**Affiliations:** ^1^Department of Neuroscience and Rehabilitation, University of Ferrara, Ferrara, Italy; ^2^Robert Hollman Foundation, Padova, Italy

**Keywords:** siblings, relations, child, adolescent, bond, vision impairment or blindness, quality of life, family relations

## Abstract

**Background:**

Vision has a crucial role in the development of social skills in early family and peer interactions. Until now, despite the large amount of literature focused on vision impairment, the number of articles dealing with the relationship between children with vision impairment and their brothers and sisters is still quite low.

**Objective:**

This systematic review aims to explore this lack by examining the existing literature on the characteristics of the sibling relationship in vision impairment in developmental age.

**Methods:**

A systematic search was performed on five databases and a total of 687 records, published between 2000 and 2025, were identified. After a selection process eight articles on the topic were finally highlighted as being eligible for analysis.

**Results:**

The characteristics of the sibling relationship mainly concern three aspects: Feelings and Behaviors, Shaped Learning in the sibling bond, and the Roles of sighted siblings in family relationships. These characteristics, reported mostly by sighted siblings, highlight the feeling-oriented and warm nature of the bond between siblings as well as the presence of some elements, which may be considered as potential risk factors for their relationship and their mental health. Overall, there is insufficient evidence to prove this due to the limited number of studies and the heterogeneity of methods applied.

**Conclusion:**

This review confirmed the paucity of literature on siblings' relationships in the field of vision impairment, highlighting many positive characteristics and some challenges that call for further research on this topic, especially with regard to the protective and risk factors for the relationship itself.

## 1 Introduction

The sibling relationship is unique because it is often the longest-lasting bond within a family, sometimes lasting a lifetime (McHale et al., 2012; Travers et al., 2020; Erdem et al., 2024). It has been described in literature as an attachment bond (Buist et al., 2013; Shenoy et al., 2024), which has characteristics of both reciprocal (e.g., peer-like) and complementary (e.g., parent-child like) interactions (Kinsley et al., 2025; Howe et al., 2022).

In childhood, the sibling bond is the first bond between peers with the peculiarity that they do not choose each other voluntarily unlike friends. Their bond spans both physical and social environments, especially in childhood. In fact, siblings share much of their time in daily interactions, making this time a unique source and context for learning, for developing socio-cognitive skills and for mutual social support (Edels et al., 2024; Howe et al., 2018, 2022; Shenoy et al., 2024).

The sibling relationship is therefore crucial in their lives, as it significantly influences their development, creating a common history and mutually molding their behavior, attitudes, and psychological functioning (Buist et al., 2013; Howe et al., 2022; Edels et al., 2024). The quality of this relationship is influenced by the individual differences of each sibling, by family dynamics, characteristics and by environmental context, which all contribute to the mental health of both siblings and, consequently, also to their quality of life (QoL; McHale et al., 2012; Incledon et al., 2015; Shojaee and Alizadeh, 2019; Hayden et al., 2023; Rawat and Malik, 2024).

The outstanding value of siblings' relationships in children is widely described in literature, even in the presence of a disability in one of the siblings (Fisman et al., 1996; Vella Gera et al., 2021; Williams et al., 2024). In the International Classification of Functioning, Disability and Health (World Health Organization, 2001), disability is a multi-dimensional concept which is defined as functioning of a person in multiple life areas and the result of interaction between the person's health condition and that person's contextual factors, specifically environmental factors and personal factors; World Health Organization, 2001). The presence of a developmental disability in one of the children can have a substantial impact on the family environment, contributing to a wide spectrum of both positive and challenging effects in the siblings (Simeonsson and McHale, 1981; Fisman et al., 1996; Rossiter and Sharpe, 2001; Veerman et al., 2023; Cooke et al., 2024). Siblings may experience feelings of love and pride and develop greater maturity, responsibility, and empathy (Shivers, 2019; Vella Gera et al., 2021; Martinez et al., 2022). They may also experience feelings of sadness, anger, jealousy, guilt, worry and embarrassment (Caliendo et al., 2020; Vella Gera et al., 2021; Martinez et al., 2022; Shenoy et al., 2024), as well as more internalizing and externalizing problems (Fisman et al., 1996; Buist et al., 2013; Martinez et al., 2022). Nonetheless this sibling relationship maintains its characteristics of uniqueness and long-life duration, profoundly influencing their lives. McHale et al. (2012) provided evidence on how siblings profoundly influence each other's development in childhood and adolescence, building on the foundations of research on siblings' relationships. This line of research draws on Adler's theory about the importance of siblings' dynamics for their psychological adjustment, on learning theories in which siblings are role models for each other in their development, and on cross-cultural perspectives that highlight what is universal in the experience of being siblings and what is instead shaped by culture and the specific context in which an individual grows up. Already in 1983, Dunn affirmed the uniqueness of the sibling relationship because it includes both complementary interactions of the child-adult relationships and reciprocal and mutually influential dynamics typical of the peer relationship. Furthermore, the high level of social interaction between siblings, which is also emotionally intense, acts as a catalyst for socio-emotional development (Dunn, 1983). The value of the intensity of the sibling bond, of the enjoyment from being together and of the time spent together has also been highlighted by Foley et al. (2025) in their study on neurodivergent twins. Shivers (2019) investigated adolescent siblings' self-reported empathy and feelings, showing greater perspective-taking ability related to positive feelings toward siblings in adolescents of individuals with autism compared to adolescents of typically developing siblings. Le Bouef and Dworkin (2021) explored how youth siblings are a context for positive development because, despite less daily communication during late adolescence and young adulthood, siblings remain an important source of support for each other; indeed, in their study, the frequency of sibling communication was also positively associated with emotional, social, and psychological well-being. Milevsky (2005) showed how in emerging adulthood social support provided by siblings is associated with psychological adjustment, in which siblings who received more sibling support scored higher on self-esteem and life satisfaction and lower on depression and loneliness than those individuals who received less sibling support. White (2001), in her panel analysis on nearly 9,000 adults who underwent two interviews approximately 5 years apart, examined four measures of the sibling relationship: proximity, contact, giving help, and receiving help. Her study showed how siblings' relationships change from the age of 18 to 85 years, showing a modest decline in contact and proximity in early adulthood and long-term stability with increased exchanges during old age. Thus, the relationship between siblings is maintained throughout the whole life and regains importance in old age, confirming its uniqueness in family relationships, mainly due to its social and emotional significance.

Despite the wide amount of literature on siblings' relationships, much less evidence is available in the field of vision impairment (VI), especially in the developmental age.

Vision impairment is mainly described as a condition of decreased visual function that interferes with the individual's ability to perform their activities of daily living (Lee et al., 2024). At the same time, through the lens of the neurodiversity paradigm, which views natural individual neurological differences and encourages their acceptance rather than considering them only as deficits (Foley et al., 2025; Cheng et al., 2025), VI can be considered a form of neurodivergence, which can be studied highlighting the role of social framing and social support as molders of development and mental health (Cheng et al., 2025).

In fact, VI significantly influences family and social interactions, as vision plays a crucial role in a child's neuropsychological, affective, and psychic development. Vision provides a scaffolding function from birth, promoting attachment relationships with caregivers through eye contact and affective-emotional attunement (Purpura and Tinelli, 2020; Battistin et al., 2024b) and fosters learning through imitation as well as through analytical and synthetic perceptive interaction with the environment (Caron et al., 2023; Baiardi and Battistin, 2022; Lo and Wang, 2024).

Indeed, all relationships within the family unit may face difficulties and challenges from the beginning because interactions and communication must be driven through the other senses, especially hearing and touch. Family dynamics change and family relationships adapt and shape themselves according to the characteristics and needs of the child with VI, who follows a neurodivergent developmental trajectory, needing more time for information processing because of their sensory impairment.

Therefore, VI affects all the areas of child development, particularly social and communicative skills, including identity recognition, emotional expression recognition, joint attention, and engagement in communication (Pinquart and Pfeiffer, 2014; Gui et al., 2023; Li et al., 2023), challenging their mental health and quality of life (Lanza et al., 2024; Langelaan et al., 2007).

The impact of contextual factors (i.e. age, gender, educational level, socioeconomical status, and social support) on mental health and QoL is well described in literature (Langelaan et al., 2007; Cheng et al., 2025). The recent systematic review by Cheng et al. (2025) stated that quite a few children with VI experience mental health issues, including anxiety, depression and low self-esteem (Li et al., 2023), and that these are connected to socio-environmental factors more than to the VI itself. Augestad (2017a) also showed a major prevalence of mood disorders in children and young adults with visual impairment, outlining how social support from family and friends as well as social activities have an important role in preventing mental health problems. Augestad (2017b) highlighted how social support and relationships play a role in increasing perceived self-esteem and self-concept, which are linked to wellbeing and psychological adjustment. Demmin and Silverstein (2020) indicated vision-specific distress, i.e. emotional reactions and distress to vision loss, as a robust predictor of depressive symptom severity as well as of anxiety, worry, withdrawal isolation in adults and older individuals. Enhanced social isolation, reduced QoL and increased risks of developing mental health problems have been shown also in children with VI (Li et al., 2024, 2023; Chadha and Subramanian, 2011).

Literature showed that the number of studies on the sibling relationship in children with and without VI is low. Kuld et al. (2020) found only one study focusing on siblings in their bibliometric map of the psychological well-being in children with vision impairment, despite the important contribution siblings can have on well-being (Jensen et al., 2022). Veldhorst et al. (2023) showed in their scoping review on the quality of life in siblings of children with vision impairment how both personal (such as prosocial behavior) and parental (such as family interaction and family functioning) factors are related to their QoL.

The aim of this study is to systematically review empirical studies on the relationship between siblings with and without vision impairment and to examine its characteristics in order to characterize it.

## 2 Systematic review method

### 2.1 Search strategy

In order to identify relevant studies concerning the characteristics of this sibling relationship, we conducted a systematic search, following the Preferred Reporting Items for Systematic Reviews and Meta-Analysis (PRISMA) guidelines (Page et al., 2021) and using five databases: Scopus, PsycINFO, Embase, PubMed, Ebsco. We also searched in Google Scholar for forward citation chasing and carried out a further hand-search in the references of all the selected articles for additional studies.

We looked for literature contributions published in the last 25 years, using a combination of the following search MeSH terms and keywords: “sibling^*^”, “visual^*^”, “vision”, “sight”, “impair^*^, “loss”, “disabilit^*^”, “vision, low” “blind^*^”, “relation^*^”, “bond^*^”, “child^*^”, “adolescent^*^”, “teen^*^”, “infant^*^”, with Boolean operators.

The systematic search started at the beginning of May and ended on June 17th, and the last update was on September 15th.

### 2.2 Selection criteria

To be included in the review, studies had to be: quantitative or qualitative or mixed research studies that focused on the sibling relationship between neurotypical siblings (NT) and neurodivergent (ND) siblings with VI in developmental age, between 0 and 18 years; only articles written in English to ensure the accuracy of data evaluation and a complete understanding of the methodological content; published in academic journals and peer reviewed.

Articles not published in peer-review journals (including pre-prints), with no full-text availability, different from research articles (such as reviews, meta-analyses, dissertations, case studies) or not written in English were excluded from our review process.

### 2.3 Study selection

The Population Intervention Comparison Outcome Study design (PICOS) protocol (Methley et al., 2014) was utilized for the content of the studies: Population: ND children or adolescents, aged 0–18 years, with visual impairment and their NT siblings; Intervention: empirical studies evaluating characteristics of the sibling relationship; Comparator: NT siblings; Outcomes: characteristics of the sibling relationship; Study type: qualitative, quantitative, mixed.

The review process followed different steps, according to PRISMA flow diagram (Page et al., 2021). Firstly, all duplicate records, identified through all the databases, were removed. Secondly, two researchers independently screened the records by relevant titles and successively by relevant abstracts. Finally, the two authors reviewed the full texts of the selected records to check whether they met the inclusion criteria. Articles that did not meet the eligibility requirements were excluded from the review.

### 2.4 Data extraction

Data were extracted independently by two researchers to ensure better accuracy. They were revised together and, having reached a consensus, combined into a final form, which was then summarized in a Microsoft Excel spreadsheet.

In particular, the following data were extracted: 1. General Information: specifically the name of the first author, the publication year and the country in which the study took place. 2. Participants' Data related to the study (the number and age range of sighted siblings; the number and age range of siblings with VI, range of VI). 3. Data about study design and methods. 4. Outcome themes and measures. 5. Findings related to outcomes.

After data extraction, qualitative findings were coded and all the results and findings were grouped according to their similarities into three main aspects.

### 2.5 Quality assessment

The articles which were finally included in the study underwent a quality evaluation, which was conducted using the Mixed Methods Appraisal Tool (MMAT, version 2018; Hong et al., 2018) applicable to both quantitative and qualitative empirical studies. This tool is based on seven questions of which two are initial screening questions and five are core quality criteria adapted to each category of study design. Two researchers independently evaluated each article scoring the seven questions with the responses “yes” or “no” or “can't tell” (not enough information reported). The inter-rater agreement was calculated as the percentage of the total number of congruent responses between the two raters, divided by the total number of questions, and found to be 94.6%. When disagreement was present, a discussion was held until a consensus was reached. Personal research was evaluated with both MMAT and the Quality Appraisal Checklist for Quantitative, Qualitative and Mixed-methods studies (QQM; Tang et al., 2025) by a researcher non included in this study in order to guarantee independent decision-making and to prevent bias in the eligibility process.

## 3 Results

**Figure 1:**
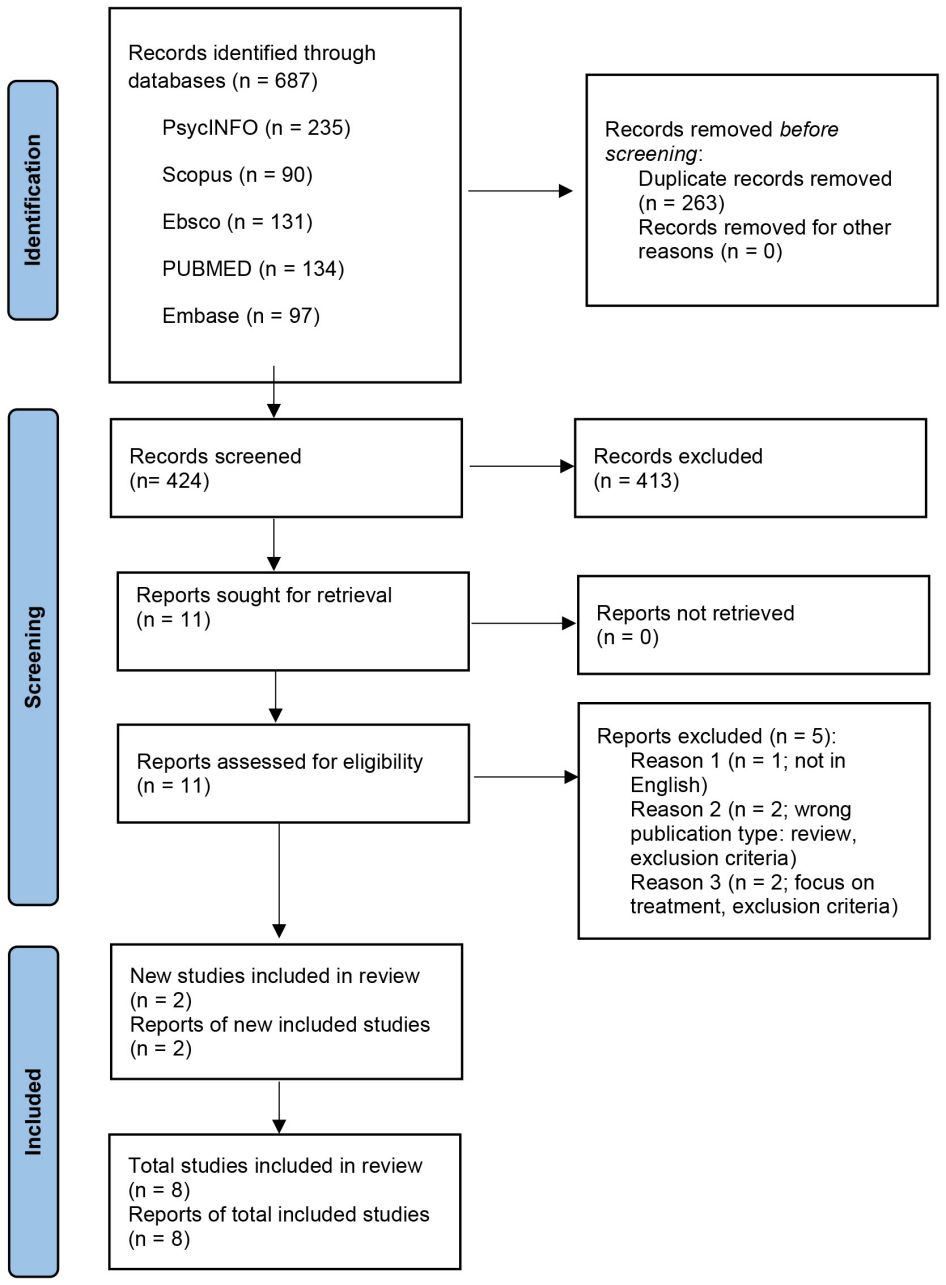
PRISMA flow diagram of the inclusion process.

A total of 687 records were initially identified through databases searches (Figure 1). After removing 263 duplicate records, 424 articles were screened first by titles and then by abstracts for relevance. During these screening phases, 413 records were eliminated for not meeting the established inclusion criteria, and 11 were selected and assessed for eligibility. During the full-text screening, five articles were excluded for the following reasons: one was not in English (*n* = 1); two because of their article type (*n* = 2); the last two were not focused on the objective of this study (*n* = 2). Only 6 articles, focusing exclusively on specific aspects of siblings' relationships connected to VI, were eligible for final analysis. Two of them are our previous studies on siblings, but we state there is no conflict of interest because this study is an extension analysis of the previous ones (Battistin et al., 2024a, 2025). Another two studies were included at the end, after a further search through references of the selected studies. Finally 8 studies underwent final analysis.

### 3.1 General characteristics of the included studies

Four out of eight identified studies used qualitative methods with self-report tools (Datta and Sabir, 2021; Lund, 2001; Battistin et al., 2024a; Erdem et al., 2024), other three (Hemati Alamdarloo et al., 2019; Ashori, 2025; Kef, 2002) used quantitative methods and another one (Battistin et al., 2025) was a mixed study. In particular Hemati Alamdarloo et al. (2019) and Battistin et al. (2025) administered the Sibling Relationship Questionnaire (SRQ; Furman and Buhrmester, 1985) and Ashori (2025) used both the Strengths and Difficulties Questionnaire (SDQ; Goodman, 1997) and the Index of Sister and Brother Relations (ISR and IBR; Hudson and Diekrs, 1997) to compare relationships in siblings of blind children to siblings of deaf and typically developing children.

Three studies (Hemati Alamdarloo et al., 2019; Ashori, 2025; Battistin et al., 2024a) focused on NT siblings while the study of Erdem et al. (2024) interviewed both siblings with and without vision impairment, as well as Battistin et al. (2025), and their parents; the study of Datta and Sabir (2021) focused on students with VI, their parents and their teachers; both the study of Lund (2001) and of Kef (2002) focused respectively on children and adolescents with VI.

The main characteristics of the included studies are presented in Table 1.

**Table 1:** Characteristics of the included studies.

**Authors (year) country**	***N* sighted siblings (SS)**	**Range years SS**	***N* visually impaired sibling (VIS)**	**Range years VIS**	**VI range**	**Study design and method**	**Outcome themes and measures**	**Findings related to outcomes**
(Erdem et al. 2024) Turkey	8 (6 families)	11–17	6	11–15	Not specified	Case-study Semi-structured interviews Inductive thematic analysis	- Diverse aspects of the relationship - Differing roles - Mutual support - Transformative experiences	Sibling relationships of children with VI were very feeling-oriented, with a philanthropic nature and an emotional ambivalence. Sighted siblings are a powerful source of support. Siblings' dyads had a limited social life with shared activities mostly home-based. Sighted siblings' excessive caregiving derived from sibling-centered parentification.
(Battistin et al. 2024a) Italy	33	7–22	29	1.5–22	From moderate VI to blindness	Qualitative cross-sectional study Semi-structured interviews Reflexive thematic analysis	- Relationships • Siblings and feelings • Parents, family members and friends - Everyday life • Personal qualities in daily life • Role in the family's everyday life - Future prospects	All sighted siblings prioritize their relationship with their sibling with VI in their lives. This relationship is characterized by unconditional love, readiness to help, but also by sorrow for the impact of VI on their QoL. Sighted siblings also learn alternative communication, in order to better help their sibling with VI. Some sighted siblings expressed a desire to spend more time with their parents. Some sighted siblings expressed concern for their siblings' future as well as an inclination toward helping professions for their future.
(Hemati Alamdarloo et al. 2019) Iran	23	10–18	-	-	Blindness (visual acuity less than 20/200 in the better eye in Iran)	Quantitative study Sibling Relationship Questionnaire (SRQ) Multivariate analysis of variance (MANOVA)	SRQ subscales: Warmth and closeness Status/power Conflict Rivalry	Significant difference in Status/power and conflict subscales compared to siblings of sighted children
(Ashori 2025) Iran	44	13–16	-	9–12	Blindness (visual acuity less than 20/200 in the better eye in Iran)	Descriptive-analytic and comparative research design. Strengths and Difficulties Questionnaire (SDQ) Index of Sister and Brother Relations (ISR and IBR) Multivariate analysis of variance (MANOVA)	SDQ subscales: - Peer relationship problems and emotional symptoms (internalizing problems) - Hyperactivity/inattention problems and conduct symptoms (externalizing problems) - Prosocial behaviors ISR and IBR: assessment of the adjustment of siblings	The adjustment and relationships in the adolescents of typically developing siblings were significantly better than the adolescents of blind siblings. The scores of externalizing and internalizing problems in the adolescents of typically developing siblings were significantly less than in the other group. There was no statistically significant difference in prosocial behavior between the adolescents of TD siblings and of blind siblings.
Battistin et al. (2025) Italy	23	7–19	22	8–14	From moderate low vision to blindness (according to ICD 11)	Mixed study: - SRQ-C (Sibling Relationship Questionnaire, Child Version). Descriptive statistic, U-Mann-Whitney test and linear regression analysis - Semi-structured interviews. Content analysis	SRQ-C: - The domain Warmth/Closeness is significantly correlated to the group category - The domain Relative Status/Power is significantly correlated to age - The sum-score of the three domains is significantly correlated both to group category and to age Semi structured interviews: - Social play and growth - Sibling versus friends - Featuring social play - Emotions and feelings	Social play has a crucial role in siblings' relationships and daily lives The great majority of siblings have a harmonious relationships quality (higher levels of Warmth/Closeness and lower in Conflict) No significant differences between NT and ND siblings in prosocial behavior ND siblings believe NT siblings have greater dominance and nurturance in their relationship Results indicate for a protective role of social play for the sibling relationship
Lund (2001) Zimbabwe	-	-	138	9–21	Not specified	Qualitative study Self-report questionnaire	Health needs Knowledge and beliefs Attitudes at home Attitudes at school Health and educational provision Information provision	Skin and eye problems Causes of albinism attributed to biological and genetic factors or to false myths and beliefs. Social problems at school Relationships between siblings appeared largely positive (help and protection), with a small minority reporting negative attitudes
Datta and Sabir (2021)	-	-	14	15–25	All levels of visual impairment (from low vision to blindness)	Semi-structured interviews. Qualitative analysis (data thematically categorized)	ND students and family relationships: perspectives of students, parents, and teachers	The majority of students reported to share a pleasant relationship with parents and siblings Parents tried to share a good relationship with their children but they are aware of the presence of factors (e.g., insensitive attitudes displayed by NT siblings) that can cause miserable family relationship According to teachers, family relationships are good when ND siblings are equally treated as their NT siblings and when family members are supportive.
Kef (2002)	-	-	316	14–24	From moderate low vision to blindness	Nationwide study and personal interviews (computer-assisted personal interviewing and computer assisted self-interviewing)	Psychological adjustment and the meaning of social support and personal networks for ND adolescents	ND Adolescents are satisfied with the social support received, even if they perceived more support from formal network members than from siblings and extended family The psychosocial adjustment of ND adolescents is reasonably good even if 18% of them have difficulties in accepting their visual impairments

### 3.2 Quality of the studies

All included studies qualified with a “yes” to the two initial screening questions regarding the clarity of the research question and whether the collected data allowed the research question to be answered. Four studies were categorized as Qualitative Datta and Sabir, (2021); Lund, (2001); Erdem et al., (2024); Battistin et al., (2024a); three as Quantitative descriptive Hemati Alamdarloo et al., (2019); Ashori, (2025); Kef, (2002), the last one Battistin et al., (2025) was categorized as a mixed study. One of these qualitative studies did not qualify for a “yes” to the question “Is the interpretation of results sufficiently substantiated by data?”—not in the interpretation of results, but rather in the data because of the extremely small number of participants interviewed for each category (ND sibling, NT sibling, parent). We did not calculate an overall score from the ratings of each criterion, as suggested in the MMAT user guide Hong et al., (2018). In summary, the overall quality of the included studies was satisfying. The accuracy and risk of bias in our research were evaluated using both MMAT and QQM. The assessment was conducted by an independent researcher who was not involved in the study and indicated a high level of quality.

### 3.3 Characteristics of the sibling relationship

All the four articles focused on characteristics of the sibling relationship and have been grouped into three main themes: Feelings and behaviors, Shaped learning in the sibling bond and Roles of sighted siblings in family relationships.

#### 3.3.1 Feelings and behaviors

The analysis of the selected articles highlighted distinct characteristics of siblings' relationships. Erdem's study (2024) described the philanthropic nature of this relationship with NT siblings more prone to keep away from conflicts, to be very indulgent, to act as confidants, and to advocate for their ND siblings. Their relationship is very feeling-oriented and is characterized by emotional ambivalence due to the intertwining between the desire to help their siblings, the desire to relate to them as with NT peers and the sense of guilt when not dedicating themselves enough to their sibling. Even in our previous study (2024a), siblings showed strong feelings such as an unconditional love and willingness to help, to the point of being their siblings' eyes in orientation and mobility situations. Both studies also described how the siblings of children with VI have feelings in common with dyads of NT children, but how they also feel sorrow for their sibling's visual disability as well as sadness and concern for their future because of their VI. Erdem et al. (2024) described also how NT children had a feeling of anxiety about their ND siblings, especially regarding their capacity to cope with social situations. Lund (2001) showed in her study that the great majority of schoolchildren did not feel treated differently by their NT siblings because of their albinism and made positive comments about their relationship. Only a small minority reported some inappropriate behaviors such as avoidance, mocking or scolding. In the study by Datta and Sabir (2021) ND siblings described a positive and congenial sibling relationship, except for a few of them who did not feel understood in their neurodiversity. Half of the parents also mentioned some inconsiderate behaviors

by NT siblings toward their ND brothers/sisters. Ashori (2025) described increased internalizing and externalizing problems in adolescents who have blind siblings, compared to those with NT siblings, attributing these difficulties to the impact of a blindness diagnosis on child socialization and family dynamics. Hemati Alamdarloo's study (2019) found no difference in feelings of warmth and closeness between siblings of blind children and those of NT children and, conversely, a statistically significant difference in the subscale of conflict. The authors explained these data as a consequence of a more difficult parent-child relationship, due to tensions and challenges, where sighted siblings sometimes misinterpret parents' behaviors, feeling more neglected than their siblings with VI. Even in our previous study (Battistin et al., 2024a), a very limited number of siblings expressed their desire to spend more time alone with parents. Our recent study on the role of social play in siblings' relationships (Battistin et al., 2025) found that both groups of siblings reported higher levels in Warmth and lower in Conflict, even if NT siblings expressed significantly higher feelings of warmth and closeness and lower of conflict than their ND siblings. We hypothesized, from the outcomes of the semi-structured interviews, that this result might be due to a less capability or will to express feelings by ND siblings and not to a less affective component. Despite this difference, the recollections from the semi-structured interviews also highlighted the depth of the sibling relationship and how this is distinguished by friendship. Conversely, Kef (2002) showed how adolescents with VI reported to receive more social support by peers than by siblings, even if the majority of them seemed satisfied with the support received from their personal networks.

#### 3.3.2 Shaped learning in the sibling bond

Another important aspect that emerged from the literature analysis is that siblings' relationships in developmental age are so intense in their everyday life experiences and feelings that the majority of their learning is mutually shaped, creating not only constructed cognitions (Erdem et al., 2024), but also, as showed in our previous study (Battistin et al., 2024a), their attitudes and future inclinations. In fact, Erdem et al. (2024) described how NT siblings constructed cognitions as a consequence of coping daily with their brother/sister's visual disability, where they normalized their sibling's impairment and at the same time empathized and thereby developed curiosity to learn more about what vision impairment is. In our previous study, we showed how NT siblings developed alternative communication skills (e.g., learning Braille) to better understand the VI “world” and to better relate to their sibling with VI. At the same time, as stated also in our previous paper (Battistin et al., 2024a), due to sharing daily life with their ND brother/sister, NT siblings developed not only a greater sensitivity toward people with disabilities, but also an inclination to choose caring professions in their future lives. This is not confirmed in all the four articles since Ashori (2025) found no statically significant difference in prosocial behavior between adolescents with NT siblings and adolescents with blind siblings as well as Battistin et al. (2025) also found no statically significant difference in prosocial behavior between NT siblings and children with VI. Kef (2002) reported how, in adolescents with VI, the sibling relationship was slightly unbalanced, with NT siblings giving more social support than receiving it.

#### 3.3.3 Roles of sighted siblings in family relationships

The sighted sibling role within family relationships was mainly explored in six out of eight studies. Both Erdem et al. (2024) and our previous study (Battistin et al., 2024a) collected the comments of NT siblings who not only expressed their willingness to help but also described their actual role as carers in everyday life. Erdem's study explained this sibling-centered parentification as arising both from their willingness to help and from parental demands. Both studies showed how this aspect was not totally negative but rather seemed to be a more spontaneous, ordinary, daily task and overall, the interviewed siblings did not feel overwhelmed by it. Battistin et al. (2025) did not found a statistically significant difference in the subscale of status/power between siblings of ND children and those of TD children, rather a significant correlation to age, with a positive mean score in NT siblings and a negative in ND ones, which suggests that ND siblings believe their NT siblings have greater dominance and nurturance in their relationship and confirmed the role of carers of NT siblings. Conversely, Hemati Alamdarloo's study (2019) found a statistically significant difference in the subscale of status/power between siblings of blind children and those of NT children. The authors explained that this data shows that children with blindness depend on all family members, not only on their parents but also on their sighted siblings who in addition to their role as siblings also act as carers. Ashori (2025) did not address this aspect specifically, even if the significant increase in internalizing and externalizing problems in the adolescents of blind siblings is also explained by stating that caring responsibilities imposed on siblings can be burdensome. At the same time siblings and other family members are described as daily sources of help for their brother/sister with VI. Lund (2001) also highlighted the role of caring, mentioned by some children with regard to aspects such as being offered additional help or protection by their siblings, especially when doing some activities outdoor in the sun.

## 4 Discussion

We investigated the characteristics of the sibling relationship in the population of siblings of children with VI through a systematic review. The analysis of the selected articles, through the lens of neurodiversity, highlighted mainly positive characteristics regarding the quality of the bond between these siblings, including their philanthropic nature, their feelings of warmth and closeness not so different from those present in NT dyads, their increased understanding of VI, its normalization and the development of alternative communicative skills, as shown in the literature also in other neurodivergences (Foley et al., 2025; Shivers, 2019; Mandleco and Webb, 2015). These positive features may also play a role as protective factors for this bond: warm relationships have been associated in literature to sibling disclosure, emotional understanding, positive interactions with more prosocial behavior and companionship (Howe et al., 2001; Buist et al., 2013; Edels et al., 2024; Kinsley et al., 2025; Foley et al., 2025). This association between the quality of the relationship and siblings' behaviors has been suggested even in other disabilities such as intellectual disabilities (Hayden et al., 2023; Travers et al., 2020) and autism (Noreen, 2021; Walton and Ingersoll, 2012; Shivers, 2019).

At the same time, as researchers, we cannot underestimate the emotional ambivalence described by Erdem et al. (2024), the statistically significant difference in the subscale of conflict reported by Hemati Alamdarloo et al. (2019), as well as some problematic behaviors reported by Lund (2001), their positively experienced carer role and an excess of responsibility, experienced as a “good behavior” which may become over time a burden (Erdem et al., 2024; Battistin et al., 2024a, 2025), that may all be potential risk factors which must be taken into consideration by caregivers and healthcare professionals. Furthermore, the feelings of sorrow and sadness for the visual condition of their brother/sister may become a risk factor for NT siblings if they do not share them with a professional with expertise, who can support them in the processing and acceptance of the situation facing them. Indeed, Ashori (2025), who focused his study not on children but on adolescents, also found a statistically significant difference in the level of internalizing and externalizing problems compared to adolescents with NT siblings, highlighting how emotional and behavioral issues may become more evident as siblings grow older. Kef (2002) also found a slightly unbalanced siblings' relationship, in which adolescents with VI giving less social support than their TD siblings and perceiving less social support from their TD siblings than from their peers. Datta and Sabir (2021), who also interviewed parents and teachers, highlighted the fundamental role of the whole family environment in the sibling relationship.

Our findings outlined how the sibling relationship in children with and without VI is mainly a positive relationship, even if complex and multifaceted. Results indicated a coexistence of potential protective and risk factors, which may develop with age and are strictly intertwined with the daily social context in which siblings live and its supportiveness. Warmth and closeness foster bonding; nevertheless, as professionals we must not underestimate all the psychological implications, burden included, associated with being brother or sister to a ND child.

This study has also some limitations: first of all, despite the large number of articles on the sibling relationship in literature, the number of articles on the sibling relationship in vision impairment is scarce. Secondly, the studies are all cross-sectional, so future studies, in particular longitudinal, are needed to explore how the sibling relationship develops with age. Thirdly, the included studies used different methods: some of them focused on and gave voice only to NT siblings, others only listened to ND children, while it should be interesting to study dyads in order to have a perspective from all the parties involved in this relationship. On this point, collecting parents' perceptions on their children's relationships, such as in two of the selected studies, could bring added value to the discussion, as well as expanding the study also to teachers or other relevant caregivers in the social environment, such as in the study by Datta and Sabir (2021). Finally, the majority of the included articles were based on data obtained from relatively small sample sizes, so studies with larger samples are necessary.

Our findings have a clinical implication in giving information to healthcare professionals on possible interventions to highlight protective factors and to prevent the onset of risk factors. We need, as clinical professionals, to work together with close family, extended family, caregivers and teachers to promote a social environment that is inclusive and sensitive to neurodiversity, highlighting siblings' strengths, listening to their worries and facilitating them when needed. At the Robert Hollman Foundation NT siblings participate in clinical and educational activities whenever possible. This is a first useful step, even if sometimes insufficient, because they also need to share their daily experiences and feelings with expert professionals and other siblings in order to strengthen all the positive aspects of their relationships and to address their difficulties in order to prevent the possible onset of mental health disorders and to improve their daily QoL (Gladyszewska-Cylulko, 2024). (Veerman et al. 2025), (2023) showed the social validity of an intervention for NT siblings using the serious game “Broodles” to help them to become more aware of their role and to be able to share their thoughts and feelings with other individuals, both professional and non-professional.

This is a priority for us as professionals, to also consider sighted siblings‘ needs to avoid impact on their mental health and on the quality of their lives. Indeed, Kirchhofer et al. (2022) showed the great value of social support for siblings of children with neurodevelopmental disorders as a protective factor to prevent negative psychosocial adjustments, as well as internalizing and externalizing behaviors. Veerman et al. (2023) also stated how siblings of children with disabilities should be supported, although there are only a few evidence-based interventions in literature on this topic. Addressing siblings' challenges facing siblings can only happen through work on the family and social environment, promoting a culture of inclusion through accurate information that values individual differences as resources and countering stigmatization.

In conclusion, this review confirmed the paucity of literature on siblings' relationships in the field of vision impairment. These findings also give a scientific contribution because they outlined some characteristics of the sibling relationship, mostly positive, but they do not allow any conclusion of evidence to be drawn due to the very limited number of included studies, with different methods and mainly small samples. The pressing need for research on this topic has been highlighted and future studies are necessary to characterize this sibling relationship in order to identify protective and risk factors for siblings' wellbeing, mental health and for the promotion of healthy family and social relationships.

## Data Availability

The raw data supporting the conclusions of this article will be made available by the authors, without undue reservation.
